# Scalpel or SABR for Treatment of Early-Stage Lung Cancer: Clinical Considerations for the Multidisciplinary Team

**DOI:** 10.3390/cancers3033432

**Published:** 2011-09-01

**Authors:** Shervin M. Shirvani, Joe Y. Chang

**Affiliations:** Department of Radiation Oncology, The University of Texas MD Anderson Cancer Center, Houston, TX 77030, USA; E-Mail: smshirvani@mdanderson.org

**Keywords:** early-stage non-small cell lung cancer, stereotactic body radiation therapy, stereotactic ablative body radiotherapy

## Abstract

Treatment options for early-stage (T1-2 N0) non-small cell lung cancer are often limited by the patient's advanced age, poor performance status, and comorbidities. Despite these challenges, stereotactic ablative radiotherapy (SABR) provides a highly effective and safe therapy for intrathoracic tumors and has become the standard of care for delivering definitive treatment in medically inoperable patients. High-quality treatment, which includes reliable immobilization, accurate tumor targeting, and precise verification of dose delivery, is essential both to achieve successful cure and to avoid debilitating toxicities. Generally, SABR is well tolerated in patients with peripherally located tumors, but even centrally or superiorly located lesions can be treated if there is adequate conformal avoidance of normal structures and/or modified fractionation to meet dose constraints. While several preliminary studies suggest that SABR is as efficacious as surgery in operable patients, results of randomized data will illuminate whether the indications for SABR can be expanded to include patients who are candidates for surgical resection. Herein, we review the rationale for using SABR and its application in treating different patient populations with early-stage lung cancer.

## 1. Introduction

The turn of the millennium represented a veritable renaissance of innovation in the field of radiation oncology. Within a span of approximately 15 years (1995–2010), a multiplicity of new technologies were not only invented but also rapidly implemented into broad clinical practice. Some of these technologies, such as three-dimensional computed tomography (CT) planning, positron-emission tomography (PET) scans, four-dimensional CT simulation, and intensity-modulated radiation therapy, have become indispensable for accurate and effective delivery of radiation therapy. Even in the company of these technologies, stereotactic ablative radiotherapy (SABR), also called stereotactic body radiation therapy (SBRT) [1], has been a particular watershed in providing a safe and definitive treatment for tumors that previously required invasive or toxic treatment for cure.

SABR delivers very high doses of radiation to the tumor target in a small number of fractions (usually five or fewer). The essence of SABR is that multiple radiation fields are made to converge on an overlap region that harbors neoplastic cells. Supplementary techniques including intensity modulation with non-coplanar beam arrangements or volumetric arcs can fine-tune the dose distribution so that the ablative doses of radiation steeply fall off beyond the intended treatment volume, thus sparing normal structures from harmful toxicity. Innovations in technology have made SABR fast and convenient; SABR can be delivered while the patient is awake, and there is no need for general anesthesia or an invasive procedure.

These characteristics have made SABR a particularly useful tool in the treatment of early-stage (T1-2 N0) non-small cell lung cancer (NSCLC), a disease that afflicts many patients who are frail and elderly. Historically, patients deemed medically inoperable have had few effective treatment options for lung cancer, even when the disease is diagnosed early. Conventionally fractionated radiation therapy delivered over the course of several weeks has resulted in disappointing local control rates of 30–50% and survival rates of only 10–30% [2,3]. One explanation for these discouraging results is that the highest biologically equivalent dose (BED) conventionally fractionated regimens could deliver before toxicities limited further dose escalation was 80 Gy, a level not sufficient for a complete tumoricidal effect. With SABR, a BED of 100 Gy or higher can be delivered safely and consistently to small tumor volumes without excessive toxicity. In contrast to the poor results of conventional fractionation, studies with SABR have resulted in local control rates of 70–98% in early-stage tumors [4-18].

The stark contrast in outcomes between conventionally fractionated radiation therapy and SABR has caused a substantial shift in the management of medically inoperable patients with early-stage lung cancer. However in order for SABR to be a high-quality treatment, there must be proper patient selection based on strong published evidence and meticulous delivery of the treatment with rigorous quality assurance steps including reliable immobilization, accurate tumor targeting, and precise verification of dose delivery. Without all of these elements, SABR has the potential to result in significant morbidity and to compromise outcomes in treatable malignancies. Herein, we review the rationale for SABR and its application in the treatment of different populations of patients with early-stage NSCLC.

## 2. Selecting Patients for SABR and Achieving High-Quality Treatment

**Figure 1.**
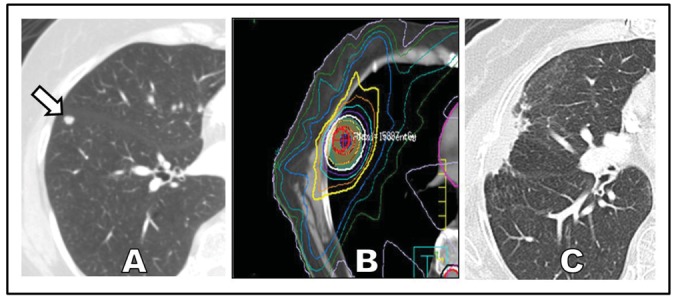
(**A**) Early-stage non-small cell lung cancer presenting as a peripheral nodule (white arrow) in a patient who was not a candidate for surgery; (**B**) Treatment with SABR to a dose of 50 Gy in 4 fractions (white isodose line); and (**C**) Surveillance computed tomography scan 3 years following treatment. The tumor has been replaced by focal consolidation.

If delivered properly, SABR can offer definitive treatment for patients with poor performance status who are newly diagnosed with early-stage NSCLC (Figure 1). Two reasons account for the enthusiastic and broad adoption of SABR over invasive surgery for this specific clinical setting. First, the median age of patients with NSCLC is 71 years, making this a disease that predominantly affects elderly patients who are likely to have coincident chronic illnesses [19]. Second, the most prevalent risk factor for lung cancer is chronic smoking, and chronic smoking carries the parallel risks of multiple systemic medical conditions, including chronic obstructive pulmonary disease [20,21], coronary artery disease [22-24], cerebrovascular disease [25], chronic renal insufficiency [26], and a number of other malignancies [27].

The combination of advanced age and medical comorbidities makes the typical lung cancer patient a risky candidate for invasive surgery. Perioperative mortality for lobectomy is typically cited to be 1–3% and depends on multiple factors, including age, sex, comorbidities, performance status, degree of dyspnea, and American Society of Anesthesiologists (ASA) score [28]. These factors have been extracted from multivariate analyses and formalized into the Thoracic Surgery Scoring System (Thoracoscore) for predicting in-hospital mortality [28-30]. Using this model. a healthy man who is younger than 55 years and is without significant comorbidities or dyspnea at baseline has a predicted mortality rate of 0.38% following lobectomy for early-stage NSCLC. On the other hand, a man who is older than 65 years and has mild to moderate comorbidities has a predicted mortality rate of 2–3%. Interestingly, the Cancer and Leukemia Group B (CALGB) trialists conducted a prospective, multi-institutional study (CALGB 39802) to elucidate the technical feasibility of video-assisted thoracic surgery (VATS) lobectomy in early-stage NSCLC and observed a 30-day mortality rate of 2.7%, which is in accordance with the Thoracoscore model [31]. The predicted mortality rate rises rapidly with decreased performance status and increased number of comorbidities, approaching 20% for the worst surgical candidates. It is in these latter high-risk patients that SABR has thus far yielded the most benefit. In such patients, peripheral lung tumors can be safely ablated with SABR without significant toxicity provided that the lesion is located away from critical structures such as the main bronchi, major vessels, trachea, heart, esophagus, spinal cord, and brachial plexus.

Prior to SABR, the radiation oncologist must address four questions in order to provide a high quality treatment: (1) Can the patient be adequately positioned and immobilized? (2) Can treatment planning account for tumor motion? (3) Will the treatment cover the intended treatment volume and spare adjacent critical structures? (4) Can the accuracy of treatment delivery be verified during treatment?

### 2.1. Positioning and Immobilizing the Patient

Positioning and immobilizing the patient are critical for treating lung tumors with SABR. Missing the tumor target can be a lost opportunity for cure because the biological characteristics of radiation preclude a “second chance” in most cases. Therefore, the position of the patient's body must be highly reproducible, and this reproducibility can be accomplished by using several reference points [32]. Typically, patients raise their arms to grasp an indexed T-shaped bar and the trunk and abdomen are placed supine on a vacuum immobilization bag that extends from the head to the pelvis and is customized to the shape of the patient's frame. For apical tumors, customized masks may be fabricated to immobilize the head and neck as well.

### 2.2. Accounting for Tumor Motion

Next, tumor motion must be accounted for; this is especially critical for lung tumors given that they exist in a continually dynamic organ. During the respiratory cycle, lung tumors can move along any directional axis (anterior-posterior, superior-inferior, or medial-lateral) and can also be stretched and/or deformed [33]. Capturing the entire tumor in the radiation field despite these multiple axes of motion is critical. Fortunately, the entire path of a tumor during a respiratory cycle can be delineated with modern four-dimensional (4-D) CT planning. The 4-D data are obtained by acquiring spatially oversampled CT images while simultaneously monitoring the patient's respiration. A collection of CT datasets is then created by either sorting or reconstructing the image data in a series of respiratory-phase bins [34]. This gives the radiation oncologist the position of the tumor and surrounding structures as a function of the respiratory phase. In turn, these images allow the radiation oncologist to customize SABR delivery by choosing one of several options, including free-breathing, respiratory-gating, or breath-holding scans, based on the tumor's characteristics and the patient's pulmonary function [32,35,36]. The utility of this technology is illustrated in the following hypothetical examples.

Patient A has a 1-cm tumor near the left apex that moves less than 5 mm in the medial-lateral axis during the respiratory cycle. Therefore, during treatment planning the tumor can be contoured at any time during the respiratory cycle and expanded by 5–8 mm to create a radiation field that will always encompass the tumor regardless of the respiratory phase. In a more challenging case, patient B is an elderly man who has a similarly sized tumor that is located 2 cm inferior to the left main-stem bronchus. The treating physician wishes to deliver the full ablative dose of radiation but fears causing unacceptable injury to the airways. To avoid this problem, the patient is instructed to hold his breath at full inspiration while wearing specialized goggles that provide visual feedback of his diaphragmatic excursion. This allows the patient to achieve the same level of inspiration at simulation for treatment planning and during daily treatments. With this maneuver, the tumor moves away from the mediastinum and left main-stem bronchus during radiation delivery.

Finally, patient C is another elderly man who has an inferiorly located peripheral tumor that is near the diaphragm. Because of chronic smoking, the patient has severe emphysema that prevents him from holding his breath long enough for delivery of a full treatment. In this case, one of two techniques can be used. One possibility is contouring the tumor at every point along the respiratory cycle and defining the radiation field as the entirety of the tumor's path (also called the internal treatment volume). This method has the disadvantage of treating more lung than otherwise required. If the additional toxicity is unacceptable, respiratory gating can be used instead. With the respiratory-gating technique, the radiation oncologist contours the tumor at a particular point during the respiratory cycle (e.g., at end inspiration), and the radiation is subsequently delivered in pulses that are synchronized to the patient's breathing such that the treatment is given only at the designated respiratory phase.

### 2.3. Covering the Treatment Volume and Sparing Adjacent Structures

The next concern is whether the tumor can be covered while normal structures are safely spared the ablative dose of radiation. With regard to tumor coverage, variations in the planning approach to dose prescription likely account for some of the differences in local control rates observed in clinical trials. As Senan *et al.* pointed out in a recent review, dose prescribed to the center of the tumor can result in an inadequate dose to the lesion's periphery and therefore worse local control [37,38]. Dose should ideally be prescribed to an isodose line outside the target, and the goal should be to deliver a biologically effective dose (BED) greater than 100 Gy to the entirety of the tumor. If the lesion is peripherally located and at least 2 cm from critical normal structures, an adequate BED can be achieved with 54–60 Gy delivered in three fractions or 48–50 Gy in four fractions prescribed to the 60%–90% isodose line. On the other hand, if the lesion is more centrally located, the dose and fractionation of 54–60 Gy in three fractions may require modification to prevent major complications. These considerations will be reviewed in more detail in section four of this review.

### 2.4. Verifying the Accuracy of Dose Delivery

Finally, the importance of geometric verification at the time of treatment cannot be overemphasized as the stakes for inaccurate delivery of SABR are especially high. Unlike traditionally fractionated radiation, SABR is unforgiving when misdirected: Missing the tumor target may allow for metastatic seeding of a once curable local disease while an inadvertently treated normal structure can lead to significant impairment and even life-threatening injury. Therefore, the advantages of this procedure are in vain if the treatment is not precisely delivered. The traditional verification strategy in conventional radiation has been to use portal films before each treatment and compare them to digitally reconstructed radiographs to ensure bony landmarks are properly aligned. Because of the low number of fractions and high dose of radiation per fraction used in SABR, use of this method lacks adequate sensitivity. Rather than use surrogate anatomic landmarks, it is strongly advised to directly visualize the lesion at the time of treatment. To that end, systems currently in use include real-time 3D imaging such as CT-on-rails and cone-beam computed tomography. Another image-guided verification strategy is the use of implanted fiducial markers at the site of the tumor. With these technologies, the position of the patient and the lesion should be accurately determined prior to the initiation of SABR treatment. As accuracy and precision improve with these technologies, the safety margin (planning treatment volume, “PTV”) of the radiation field can be decreased to assist in sparing of critical normal tissues.

If the above technical procedures are instituted in a formalized setting with strict quality assurance measures, most patients with early-stage lung cancer can expect excellent treatment, recovery, and eradication of their lesion with SABR. Finally, it should be noted that the external credentialing required for participation in clinical trials is one mechanism to ensure that institutions meet these standards.

## 3. Evolving Paradigms for the Treatment of Early-Stage NSCLC

Multiple prospective studies of SABR have consistently demonstrated high control of the primary lesion (Table 1), and lessons from early experiences with SABR in North America culminated in the Radiation Therapy and Oncology Group (RTOG) Trial 0236, which accrued 59 patients between 2004 and 2006. In this trial, early-stage tumors were treated to 60 Gy in 3 fractions (54 Gy with heterogeneity correction). With this regimen only one local failure within 2 cm of the original lesion and three additional failures within the involved lobe were observed, resulting in an actuarial local control rate of 98.0% and regional control rate of 90.7% at 3 years [4]. In light of these excellent results, standard of care for the inoperable patient has shifted from palliation and supportive care to SABR. The positive effect on public health by this shift has been confirmed in at least one population-based cancer registry. After the introduction of SABR in a Dutch province, a decline in the proportion of untreated elderly patients with early-stage lung cancer was observed along with improvement in the median survival of such patients [39].

Are there other patient populations who should be treated with SABR? One patient population who would probably benefit is those who have been previously treated with conventionally fractionated radiation and have experienced local recurrence or a new primary. In this setting, re-irradiation with SABR has been shown to provide greater than 90% in-field local control with manageable toxicity [40]. The more controversial and timelier question is whether the patient population for whom SABR is the first-line treatment should be expanded to operable patients with early-stage lung cancer. Expressed differently, given that some inoperable patients in the SABR protocols had outcomes as good as or better than the historical outcomes in their operable counterparts, should the scope of SABR reach beyond those who cannot safely undergo surgery?

The motivation for expanding the indications for SABR is that while surgical resection is a mature and effective therapeutic approach with acceptable toxicity, nonetheless it is not risk free. A systematic review of anatomic resection techniques suggests that the overall complication rate of VATS lobectomy is 16.4% and thoracotomy lobectomy is 31.2% [41]. The most common complications are atrial fibrillation, postoperative pneumonia, and persistent air leakage from a chest tube. Another important phenomenon when considering surgery is the rising prevalence of patients requiring antiplatelet medications. Given the high correlation of lung cancer and cardiovascular disease predicated on tobacco use, a growing number of patients with lung cancer at presentation will be on aspirin or thienopyridine therapy (e.g., clopidogrel) to reduce risk of coronary events or to prevent thrombosis after placement of a coronary stent [23,42]. Discontinuation of aspirin or thienopyridine therapy before surgery can result in myocardial infarction or even death, and the American Heart Association recommends that patients who underone placement of a drug-eluting stent defer elective surgeries for up to a year in order to complete antiplatelet therapy [43-46]. Historically, patients in this clinical situation have had to make a choice between the two unpalatable options of proceeding with surgery at the risk of in-stent thrombosis or deferring surgery until thienopyridine therapy is complete and risk the possibility of cancer progression in the meantime.

With the above considerations in mind, SABR presents an attractive treatment option for some patients who are deemed operable. Unfortunately, as yet there is no published randomized data directly comparing SABR with surgery for operable patients. The ROSEL trial in the Netherlands sought to randomize stage IA patients into two treatment arms, but this trial closed early due to poor accrual [47]. Another trial sponsored by Accuray Inc. (the international STARS trial) is currently accruing patients, but final results will likely not be published until 2014 [48]. The American College of Surgeons Oncology Group (ACOSOG) has opened a trial (Z4099) comparing SABR and sublobar resection for high-risk operable patients [49]. Finally, Varian Inc. has approved support for a randomized study to compare surgery and SABR.For now, available evidence suggests that SABR may turn out to be a feasible option in patients able to undergo lobectomy. For instance, a retrospective review of medically operable patients in a multi-institutional study in Japan yielded 5-year local recurrence and overall survival rates of 8.4% and 70.8%, respectively, among medically operable patients treated with SABR with a BED greater than 100 Gy [5]. These are roughly comparable to the 5-year outcomes observed in the lobectomy arm of the North American Lung Cancer Study Group (LCSG) 821 trial that compared wedge resection and lobectomy (local recurrence and overall survival of 6% and 70%, respectively) [50].

A more direct comparison, albeit also retrospective, of surgery and SABR was published by investigators from William Beaumont Hospital group [11]. Between 2003 and 2008, patients categorized as borderline operable owing to cardiopulmonary comorbidities were treated with either SABR or sublobar (wedge) resections at that institution. Review of this center's experience demonstrated that SABR and sublobar resection had the same rate of distant metastases and cause-specific survival, but a nonsignificant trend was observed for a decreased risk of local and regional recurrence in the SABR group. A similar propensity matched analysis of SABR and surgery reiterated equal rates of local recurrence and disease-specific survival between the two approaches [51].

Finally, outcomes of two single-arm prospective studies of operable patients treated with SABR have recently been reported by investigators from Japan and the Netherlands. Nagata *et al.* reported initial outcomes from the Japanese Clinical Oncology Group (JCOG) 0403 trial, a prospective phase II study in which stage IA operable patients received 48 Gy in four fractions. The 65 patients in this study were elderly (median age, 79 years) but were generally in good health (performance status 0–2, PaO2 ≥ 60 torr, FEV1.0 ≥ 700 mL) and deemed operable by thoracic surgeons. With a median follow-up of 45.4 months, the 3-year overall survival rate was 76.0%, and the 3-year local progression-free survival rate was 68.5%. Grade 3 toxicities included chest pain (1.5%), dyspnea (3.1%), hypoxia (1.5%), and pneumonitis (3.1%). No grade 4 or 5 toxicities were observed [17].

Similarly, the Dutch researchers' single-institution prospective study followed “potentially operable” patients who underwent SABR in lieu of surgery [18]. In this slightly younger patient group (median age, 76 years), the 3-year local control and overall survival rates were 93.0% and 84.7%, respectively. Reported toxicity was also mild, with severe (grade ≥ 3) radiation pneumonitis and rib fractures occurring in 2% and 3% of patients, respectively. Although these data are not as compelling as randomized evidence, it is hard to imagine that these potentially operable patients would have had better outcomes had they undergone lobectomy. A third single-arm study of operable patients is being conducted in the United States by the RTOG, and its results are eagerly awaited [52]. In light of the many questions that remain to be answered regarding optimal patient selection for SABR, we recommend that patients be enrolled in clinical trials whenever possible.

One other issue in the evolution of early-stage lung cancer treatment merits attention. The introduction of systemic chemotherapy and molecularly targeted agents for patients with early-stage lung cancer has the potential to further complicate the choice between surgery and SABR. There is already some enthusiasm for using such agents in certain populations, and a vast effort is underway to identify biological markers for subtypes of lung cancer, including tumors with a high propensity for distant metastases regardless of tumor stage [53-56]. If systemic agents are introduced for patients with early-stage disease, important questions regarding local control must be addressed. Will these agents sterilize the involved lobe of the lung and regional nodes, obviating the need for lobectomy and favoring SABR for local control? Or instead will these agents be better at preventing distant failure, making the need to address regional structures with surgery even more critical? Ultimately, the way in which systemic agents will tip the balance between SABR and anatomic resection will require empirical study with formal clinical trials.

## 4. SABR Toxicities and Challenges

Despite showing great promise in prospective studies, pitfalls remain regarding treatment toxicities and patient management during follow-up. With regard to the former, the ablative doses of radiation used in a course of SABR can cause considerable damage to normal tissues, resulting in severe complications and diminished quality of life. Therefore, appropriate anatomical selection of candidates is crucial if the advantages of SABR are to be meaningful for the individual patient. For lesions located in the periphery of the lung but away from the chest wall, the use of very high doses of radiation are more permissible because the lung is a parallel organ and can generally tolerate the loss of functional alveolar units immediately around the neoplasm. However, grade 3–4 toxicity rates approached 30% in early studies even among this group with favorably located tumors, underlining the need for technical expertise and quality assurance for safe treatment; with improvements in technique, grade 3–4 toxicity rates now occur in the range of 0–15% for these anatomically favorable patients [4,9,14,15,57]. Radiation pneumonitis is the most frequent toxicity observed, and investigators have recommended limiting the volume of the lungs receiving 20 Gy (V20) to avoid this adverse effect [56]. The degree of limitation is dependent on the fraction size, and clinical trials have used 5–20% of the total lung volume as the threshold [58].

If the lesion is centrally located, SABR can result in severe injury to mediastinal structures, abrogating its advantages over surgery. A phase II study of 70 patients with both peripherally and centrally located tumors treated to 60–66 Gy (without heterogeneity correction) in three fractions found 2-year freedom from severe toxicity rates of only 54% for patients with centrally located tumors compared to 83% in patients with peripherally located tumors [59]. These toxicities included pleural effusion, pneumonia, and a decline in pulmonary function. Even more sobering, four of six deaths were likely related to treatment, andthese deaths occurred in patients with centrally located tumors. In light of these results, the authors of the study recommended that this dose-fraction regimen not be used in patients with tumors near the central airways. Other very rare but devastating toxicities of central structures include tracheal/bronchial injury, esophageal ulceration, and spinal cord myelopathy [60,61]. With appropriate patient selection, treatment planning, and adherence to dose constraints, these events should not occur with measurable frequency.

Tumors near the chest wall and brachial plexus also have the potential for grave injury. In a series of 36 patients with tumors at the apex treated with a three-fraction regimen to a median dose of 57 Gy, the rate of brachial plexopathy was 19.4% [62]. Patients experienced neuropathic pain, arm weakness, and, inone case, extremity paralysis. Those receiving more than 26 Gy to the brachial plexus had the highest risk of neuropathy (46%), so this value is now typically cited as brachial plexus' maximum tolerable dose. Rib fractures are another potential complication of SABR, and two studies have examined their incidence. Nine fracture events occurred among 42 patients in the first study, and five events occurred among 60 patients in the second study [63,64]. With regard to chest wall pain, a large institutional series of 265 patients with tumors within 2.5 cm of the chest wall reported a 17% rate of chronic chest wall pain [65]. Notably, obesity and diabetic state were associated with the development of chronic pain in this study. Because 30 Gy appears to be the inflection point at which incidences of chest wall pain and rib fracture increase, the volume of the chest wall that receives this dose should be limited [64,65].

Examples of organ dose limitations used in major trials in North America and Europe are presented in Table 2, but it should be noted that these values will likely change as clinical experience with SABR increases [47,52,66]. Moreover, the incidence of the aforementioned toxicities depends on the total dose and fraction size of the SABR treatment. By modulating these parameters, one can lower rates of adverse effects and achieve greater flexibility in the regions that are amenable to SABR. For example, a series of 27 patients with centrally and superiorly located tumors was treated with an alternative regimen of 50 Gy in four fractions for just this purpose. Complication rates were modest given the tumors' central location. Four patients (14.8%) developed grade 2 pneumonitis, three patients (11.1%) developed grade 2–3 skin toxicity and/or chest wall pain, and one patient developed brachial plexus neuropathy [67]. Another regimen used for centrally located lesions with reassuring rates of observed toxicities administered 70 Gy in 10 fractions [15]. The ideal dose and fractionation scheme is a topic of active clinical investigation, and the RTOG is currently conducting two trials (RTOG 0813 and RTOG 0915) to clarify the ideal dose and fractionation for SABR [68-70].

A different kind of challenge in the use of SABR is the delineation of proper follow-up after treatment has occurred. Parenchyma changes such as fibrosis and persistent radiation pneumonitis can occur following SABR, and the resultant radiographic appearance on surveillance CT imaging can run the gamut from diffuse consolidation and ground-glass opacities to focal consolidation and scarring. In some cases, focal consolidation during follow-upis hard to distinguish from tumor recurrence (Figure 1C). This wide spectrum of radiographic changes can render tools like the RECIST criteria insufficient for evaluation of local response. Moreover, PET as a means to distinguish postradiation effects from viable tumor is problematic, as treated regions can have persistent [^18^F ] fluorodeoxyglucose avidity for up to a year following SABR [72,73]. More recent studies suggest that specific findings on PET such as a high post-SABR standardized uptake volume (>5) more than 3 months after SABR can better select the best patients for biopsy to confirm recurrence [74]. Despite these diagnostic challenges, a lack of clear progression of a tumor following treatment is a positive sign, and an experienced radiologist familiar with post-SABR effects should be able to distinguish between SABR effects and tumor recurrence. Identifying recurrence is important as emerging studies suggest that salvage surgery following SABR may be feasible [75]. Future studies are expected to illuminate these controversial areas and provide evidence-based guidance for surveillance and, if necessary, salvage therapy after SABR.

## 5. Conclusions

The early-stage lung cancer patient can present a therapeutic challenge due to advanced age, frail performance status, and comorbidities arising from tobacco use. For patients who are medically inoperable, SABR has become the standard of care for achieving a high rate of local control and overall survival. Toxicity is minimal with peripherally located tumors, but centrally or superiorly located lesions can also be treated with a modified dose and fractionation. Proper technique that includes reliable immobilization, accurate tumor targeting, and precise verification of dose delivery is critical for safe, effective, and ethical use of this technology. While several preliminary studies suggest that SABR could be as efficacious as surgery in the treatment of operable patients, the impetus to preserve or change practice patterns will likely depend on the publication of ongoing randomized trials. In the meantime, lobectomy remains the standard of care for patients with early-stage (T1-2 N0) NSCLC if they are in good health and have adequate pulmonary function. Whether future physicians unsheathe SABR instead of a scalpel for these patients remains to be seen.

## Figures and Tables

**Table 1.** Selected Prospective Studies of SABR for Early-Stage Non-small Cell Lung Cancer.

**Trial**	**Stage**	**Dose and fractionation**	**Local control**	**Overall survival**
Timmerman [34]	T1-T2 N0	60 Gy in 3 fractions	98.0% (3 years)	72.0% (2 years)
Fakiris [6]	T1-T2 N0	60-66 Gy in 3 fractions	88.1% (3 years)	42.7% (3 years)
Nagata [9]	T1-T2 N0	48 Gy in 4 fractions	94.0% (3 years)	T1: 83.0%; T2: 72.0% (3 years)
Ricardi [12]	T1-T2 N0	45 Gy in 3 fractions	87.8% (3 years)	57.1% (3 years)
Xia [15]	T1-T2 N0	70 Gy in 10 fractions	95.0% (3 years)	78.0% (3 years)
Chang [16]	T1-2 N0	50 Gy in 4 fractions	98.5% (2 years)	78.2% (2 years)
Nagata [17]	T1 N0 (operable)	48 Gy in 4 fractions	68.5% (3 years)	76.0% (3 years)
Senan [18]	T1-T2 N0 (operable)	60 Gy in 3, 5, or 8 fractions	93.0% (3 years)	84.7% (3 years)

**Table 2.** Dose Constraints Used in Major North American, European, Asian and International Trials. Limits represent point doses unless otherwise specified.

**Organ at risk**	**RTOG** **1** **0618 (3 fractions)**	**Dutch ROSEL** **2** **trial (3 fractions)**	**Dutch ROSEL trial (5 fractions)**	**International STARS** **3** **trial (4 fractions)**	**JCOG** **4** **0403**
Spinal cord	≤18 Gy	≤18 Gy	≤25 Gy	20 Gy ≤ 1 cc	≤25 Gy
				15 Gy ≤ 10 cc	
Lung	V20 ≤ 10%	V20 ≤ 5-10%	V20 ≤ 5-10%	V20 ≤ 20%	V15 ≤ 25%
				V10 ≤ 30%	40 Gy ≤ 100 cc
				V5 ≤ 50%	MLD ≤ 18 cc
Esophagus	≤27 Gy	≤24 Gy	≤27 Gy	35 Gy ≤ 1 cc	40 Gy ≤ 1cc
				30 Gy ≤ 10 cc	35 Gy ≤ 10 cc
Brachial plexus	≤24 Gy	≤24 Gy	≤27 Gy	Point ≤ 40 Gy	Not limited
				35 Gy ≤ 1 cc	
				30 Gy ≤ 10 cc	
Heart	≤30 Gy	≤24 Gy	≤27 Gy	40 Gy ≤ 1 cc	48 Gy ≤ 1 cc
				35 Gy ≤ 10 cc	40 Gy ≤ 10 cc
Trachea	≤30 Gy	≤30 Gy	≤32 Gy	35 Gy ≤ 1 cc	40 Gy ≤ 10 cc
				30 Gy ≤ 10 cc	
Bronchi	≤30 Gy	≤30 Gy	≤32 Gy	40 Gy ≤ 1 cc	40 Gy ≤ 10 cc
				35 Gy ≤ 10 cc	
Skin	≤24 Gy	Not limited	Not limited	40 Gy ≤ 1 cc	Not limited
				35 Gy ≤ 10 cc	

1 Radiation Therapy Oncology Group 0618 [52].

2 Randomized clinical trial of surgery versus radiosurgery in patient with stage IA NSCLC who are fit to undergo primary resection [47].

3 Randomized study of lobectomy versus cyberknife for operable lung cancer [48].

4 Japanese Clinical Oncology Group 0403 [71].
